# Oxidative stress in ARDS: mechanisms and therapeutic potential

**DOI:** 10.3389/fphar.2025.1603287

**Published:** 2025-06-26

**Authors:** Fengyun Wang, Ruiqi Ge, Yun Cai, Mingrui Zhao, Zhen Fang, Jingguo Li, Chengzhi Xie, Mei Wang, Wanyue Li, Xiaozhi Wang

**Affiliations:** ^1^ Department of Critical Care Medicine, Second Affiliated Hospital of Hainan Medical University, Haikou, China; ^2^ Department of Critical Care Medicine, Zhongnan Hospital, Wuhan University, Wuhan, Hubei, China

**Keywords:** acute respiratory distress syndrome (ARDS), acute lung injury (ALI), oxidative stress, reactive oxygen species (ROS), inflammation

## Abstract

Acute respiratory distress syndrome (ARDS) is a life-threatening condition characterized by acute lung inflammation, increased vascular permeability, and hypoxemic respiratory failure. Oxidative stress, driven by excessive reactive oxygen species (ROS), is a key contributor to ARDS pathogenesis, causing cellular damage, inflammation, and alveolar-capillary barrier disruption. This review elucidates the mechanisms of oxidative stress in ARDS, focusing on ROS production via NADPH oxidase (NOX) and mitochondria, which activate pathways like NF-κB and MAPK, promoting pro-inflammatory cytokine release. ROS-induced lipid and protein peroxidation, endothelial dysfunction, and programmed cell death (PCD), including apoptosis, pyroptosis, and ferroptosis, exacerbate lung injury. In COVID-19-related ARDS, SARS-CoV-2 spike protein amplifies mitochondrial ROS, worsening outcomes. Antioxidant therapies falter due to non-specific ROS suppression, patient heterogeneity (e.g., GSTP1 polymorphisms), and poor bioavailability. We propose a model where oxidative stress drives ARDS stages—early alveolar injury and late systemic dysfunction—suggesting targeted therapies like endothelial-specific nanoparticles or ferroptosis inhibitors. Precision medicine using biomarkers (e.g., mtDNA) and gender-specific approaches (e.g., estrogen-Nrf2 regulation) could enhance outcomes. This review bridges mechanistic gaps, critiques therapeutic failures, and advocates novel strategies like mitochondrial-targeted therapies to improve ARDS management.

## Introduction

Acute respiratory distress syndrome (ARDS) is a lethal disease that frequently leads to respiratory failure in severely ill individuals, characterized by the sudden emergence of non-cardiogenic lung edema, hypoxemia, and the requirement for mechanical ventilation support (Fan et al., 2017; Wick et al., 2024). The etiologies of ARDS comprise factors such as infections (including bacterial and viral pneumonia), trauma, reflux aspiration, inhalation of noxious substances, acute pancreatitis, etc. (Gorman et al., 2022; Qadir et al., 2024).

The term oxidative stress describes a situation where reactive oxygen species (ROS) accumulate excessively, exceeding the protective capacity of antioxidants, and ultimately causing damage to cells, tissues, and organs (Sies, 2015). The group of ROS consists of superoxide anion (O_2_^−^), hydrogen peroxide (H_2_O_2_), and hydroxyl radicals (OH·), which compromise cellular integrity and functionality by oxidizing key macromolecules such as lipids, proteins, and DNA (Circu and Aw, 2010). Oxidative stress is essential in triggering and exacerbating a series of diseases, including cardiovascular conditions, diabetes, neurodegenerative issues, and respiratory disorders (Aruoma et al., 2006).

Major factors contributing to oxidative stress within the lungs consist of air contaminants, cigarette consumption, infectious agents, mechanical ventilation usage, and localized inflammatory reactions (Bargagli et al., 2009; Guatura et al., 2000). Oxidative stress initiates inflammation, leads to cell death, causes tissue injury, and promotes remodeling by activating different signaling pathways (including NF-κB and MAPK) and immune cells (like neutrophils, macrophages, and T-cells) (D'autreaux and Toledano, 2007).

Given its pervasive impact, understanding oxidative stress in ARDS is critical to unraveling its pathophysiology. During the onset and progression of ARDS, oxidative stress plays a pivotal role. Numerous research efforts indicate that oxidative stress not only harms alveolar epithelial and endothelial cells, disrupting the alveolar-capillary barrier, but also intensifies inflammation by triggering pro-inflammatory cytokines and immune cell activation (Tasaka et al., 2008; Karapetsa et al., 2013). This review proposes a model where oxidative stress drives a temporal cascade—early alveolar injury escalating to late immune dysregulation and multi-organ dysfunction—guiding targeted therapeutic strategies. The objective of this review is to discuss the key mechanisms underlying oxidative stress in ARDS, especially its effects on lung injury, the inflammatory response, and immune system modulation. Additionally, this review aims to assess oxidative stress as a potential therapeutic target for ARDS and review the practicality and clinical use of antioxidant therapies, providing new insights and approaches for the prompt identification and treatment of ARDS.

## Pathophysiology of ARDS

The pathophysiology of ARDS, characterized by acute lung injury, leads to severe respiratory distress and hypoxemia, driven by oxidative stress and inflammation. These mechanisms, detailed below, disrupt the alveolar-capillary barrier, causing pulmonary edema and impaired gas exchange, which translate into clinical manifestations discussed in later sections (Fan et al., 2017; Force et al., 2012; Han and Mallampalli, 2015).

### Cellular and molecular mechanisms of ARDS

The underlying processes of ARDS are driven by the interaction of multiple cell types and various molecular mechanisms. Critical cellular and molecular aspects involve.

#### Alveolar epithelial cell injury and repair

During ARDS, the alveolar epithelial cells are typically the first to sustain injury. The formation of ROS and the secretion of inflammatory factors contribute to PCD, such as apoptosis, pyroptosis, and ferroptosis of these cells, worsening lung damage and compromising the barrier function (Millar et al., 2016). The stimulation of cytokines like IL-1β and TNF-α leads to the death of cells (Galani et al., 2010).

#### Capillary endothelial cell injury

Injury to the endothelial cells lining the capillaries is a vital element in ARDS. This type of injury is significantly related to oxidative stress, as ROS influence endothelial cells directly, activating pathways like NF-κB that promote inflammation and modify vascular permeability (Sarada et al., 2008). This increases the permeability of the blood vessels, allowing proteins and fluids to escape from the bloodstream into the lung interstitial space, which causes pulmonary edema (Vassiliou et al., 2020).

#### Inflammatory response

Inflammation is fundamental to the beginning and progression of ARDS. The early stages of this condition heavily depend on immune cells, such as resident alveolar macrophages and recruited neutrophils. Immune cell activation is significantly influenced by ROS, which activate signaling pathways, resulting in a heightened release of pro-inflammatory cytokines, including IL-8 and TNF-α, thus aggravating inflammation in the lungs (Lee and Yang, 2012; Rabadi et al., 2016).

#### Cytokines and chemokines

The inflammatory response associated with ARDS is primarily driven by the release of various cytokines and chemokines. Notable cytokines, such as TNF-α, IL-1β, and IL-6, encourage the influx of inflammatory cells and increase the permeability of blood vessels, thereby leading to the disruption of the alveolar-capillary barrier (Tang et al., 2014; Bry et al., 2007). Also, chemokines like IL-8 enhance the recruitment of neutrophils to the lung area, further escalating the inflammatory response (Allen and Kurdowska, 2014).

Oxidative stress, via ROS from NADPH oxidase (NOX) and mitochondria, disrupts alveolar-capillary tight junctions (e.g., Zonula occludens) and increases vascular permeability, causing pulmonary edema (Mittal et al., 2014; Li D. et al., 2020; Wang et al., 2012). It amplifies inflammation by recruiting neutrophils and macrophages, perpetuating a vicious cycle of lung injury (Sapoznikov et al., 2019; Sun et al., 2023; Kellner et al., 2017).

In ARDS, oxidative stress drives dysregulated immune responses, particularly via innate immunity. ROS-induced HMGB1 release recruits neutrophils and macrophages, amplifying inflammation (Li R. et al., 2020; Nakamura et al., 2009). Adaptive immunity is also affected, with T-cell glycolysis/OXPHOS imbalance increasing ROS and cytokine production (Speer et al., 1984).

## Sources of oxidative stress

### Main sources of ROS: NOX and mitochondrial dysfunction

Oxidative stress is predominantly connected to the excessive generation of ROS, such as superoxide anions (O_2_¯), hydrogen peroxide (H_2_O_2_), and hydroxyl radicals (OH·). Key enzymatic and organellar sources include NADPH oxidases (NOX) and mitochondria.

**FIGURE 1:**
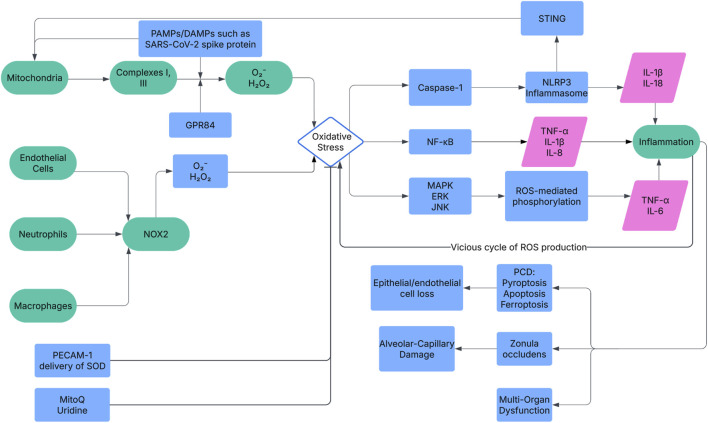
The mechanism network of oxidative stress in ARDS pathogenesis. ROS from NOX and mitochondria activate NF-κB, MAPK, and NLRP3 pathways, upregulating cytokines (e.g., IL-1β). This drives alveolar damage (ZO disruption), inflammation, PCD (apoptosis, pyroptosis, ferroptosis), and multi-organ dysfunction. Key molecules include NOX2, NF-κB, and Gpx4. Therapeutic targets like PECAM-1 nanoparticles and mitoQ aim to mitigate ROS effects. NOX, NADPH Oxidase; NF-κB, Nuclear Factor-κB; MAPK, Mitogen-Activated Protein Kinase; NLRP3, NOD-like Receptor Protein 3; IL-1β, Interleukin-1β; ZO, Zonula Occludens; PCD, Programmed Cell Death; Gpx4, Glutathione Peroxidase 4; PECAM-1, Platelet Endothelial Cell Adhesion Molecule-1.

NADPH oxidase (NOX) and mitochondria are primary ROS sources in ARDS, driving early alveolar damage and late systemic dysfunction (Figure 1). NOX is a key enzyme involved in the synthesis of ROS, specifically by forming O2¯ through the transfer of electrons (Vermot et al., 2021). Recent studies, including those utilizing Nox2^−/−^ mice models {Hook, 2019 #668}, have further elucidated the role of NOX2 in neutrophil-mediated ROS production, demonstrating that its activation by GPR84 exacerbates LPS-induced ALI through Lyn, AKT, and ERK1/2 signaling pathways, highlighting a specific molecular cascade driving oxidative stress in ARDS (Wang et al., 2023). Mitochondrial ROS, generated at Complexes I/III, escalate under hypoxia, contributing to cell death (Stowe and Camara, 2009; Puri and Naura, 2022). Recent research has shown that cold-stimulated bronchial epithelial cell-derived exosomes (CS-BECs-exo) exacerbate sepsis-induced ALI by increasing mitochondrial ROS via the HMGB1/RAGE/Nrf2/HO-1 pathway, suggesting a novel extracellular vesicle-mediated mechanism of mitochondrial oxidative stress in ARDS (Wang et al., 2025). In the context of COVID-19 ARDS, the SARS-CoV-2 Spike protein has also been shown to induce mitochondrial ROS, contributing to ALI pathogenesis (Zhang et al., 2022). Studies also demonstrate that NLRP3 deletion (Fukumoto et al., 2013) and ASK1 knockout (Fukumoto et al., 2016) attenuate hyperoxia-induced ALI by suppressing ROS-mediated inflammasomes.

#### Activation of neutrophils

Neutrophils are critical in lung inflammation, as they migrate to affected areas and generate ROS as part of their antimicrobial defense mechanism. However, this process can also cause tissue damage and intensify inflammation. In ARDS, neutrophils are actively involved in the inflammatory response and produce high levels of ROS through their NOX activity (Abraham, 2003). Additionally, neutrophil-specific proteins like CD177 have been shown to enhance ROS production and neutrophil extracellular trap (NET) formation, contributing to ARDS progression via NLRP3 inflammasome activation, offering a novel mechanistic link between neutrophil activity and oxidative stress (Li et al., 2024).

## The mechanism of oxidative stress in ARDS

The impact of oxidative stress on ARDS is mainly characterized by its role in worsening the damage to the alveolar-capillary barrier, provoking inflammatory responses, and resulting in cell death, which collectively contribute to the initiation and evolution of ARDS. Oxidative stress directly damages alveolar epithelial and capillary endothelial cells, compromising alveolar-capillary barrier integrity and triggering inflammatory cascades (Figure 1). Key mechanisms include.

### Oxidative stress-induced programmed cell death

Oxidative stress triggers multiple forms of programmed cell death (PCD), including apoptosis, pyroptosis, and ferroptosis, amplifying ARDS severity. Apoptosis, driven by ROS-induced mitochondrial dysfunction and DNA damage, disrupts alveolar epithelial and endothelial cell integrity (Tamada et al., 2020). Pyroptosis, mediated by the STING-NLRP3 axis, is activated by cytosolic mitochondrial DNA and cGAS, promoting macrophage death in LPS-induced ALI (Ning et al., 2020). Ferroptosis, linked to Gpx4 downregulation by BACH1, increases lipid peroxidation and lung necrosis (Amaral et al., 2024). These pathways, regulated by NLRP3/STING and GPX4, collectively worsen barrier dysfunction and inflammation (Kang et al., 2022; Wang X. et al., 2022). P2X7 deletion (Galam et al., 2016) and resolvin D1 treatment (Cox et al., 2015) reduce hyperoxia-induced PCD, highlighting targeted ROS suppression. Disruption of the alveolar-capillary barrier: ROS disrupt the alveolar-capillary barrier by damaging epithelial and endothelial cells, altering junction proteins, and increasing vascular permeability, leading to pulmonary edema (Veit et al., 2015; Di et al., 2016). Ripk3 exacerbates this via AMPK/Drp1/mPTP-mediated necroptosis (Zhao et al., 2025).

Promotion of inflammatory response: ROS-driven inflammation, via NF-κB and MAPK, promotes cytokines (TNF-α, IL-1β) that recruit neutrophils, perpetuating a feedback loop of ROS production and tissue damage (Lee and Yang, 2012; Han et al., 2013; Dada and Sznajder, 2011). L-10-regulated Fth1hi neutrophils exacerbate this cycle in ALI (Wang K. et al., 2022).

### Impact of oxidative stress on immune response in ARDS

The presence of oxidative stress in ARDS is linked to both cellular and tissue injury, as well as an enhanced immune response through the regulation of immune cell activities.

Excessive ROS production can cause immune cells to become overly activated, and in some instances, these cells may incur harm, which can further aggravate the progression of ARDS (Kellner et al., 2017). Neutrophils and macrophages, key components of the immune system, are heavily impacted by oxidative stress. The presence of ROS initiates signaling pathways within these cells, resulting in the production of inflammatory cytokines and chemokines (for instance, IL-8 and MCP-1) and boosting cellular migration, thus worsening the local immune response (Speer et al., 1984; Zeng et al., 2019).

The interplay between immune metabolism and oxidative stress is also critical; for example, T cell metabolic reprogramming, such as shifts in glycolysis and oxidative phosphorylation (OXPHOS), can significantly influence ROS generation and the inflammatory cascade in ARDS (Li et al., 2025). Additionally, the modulation of T cell activity by ROS can significantly influence the adaptive immune response. For instance, in experiments with *Streptococcus* pneumoniae lung infections, SOD3-deficient mice demonstrated considerably reduced levels of the monocyte chemokine CCL-2 and the cytokines IL-23, IL-1β, and IL-17A. This reduction was associated with a reduction in γδ T cells expressing IL-17A within the lung tissue (Anthony et al., 2020). Moreover, myeloid PTEN deficiency has been shown to regulate the YAP-Nrf2 axis in macrophages, reducing oxidative stress and inflammation by inhibiting GSK3β and MST1 interactions, offering a protective mechanism against LPS-induced ALI (Liu et al., 2024). Furthermore, Bach1 deficiency ameliorates radiation pneumonitis by upregulating TFAM, enhancing mitochondrial function, and reducing macrophage-driven inflammation and oxidative stress, suggesting a protective immune-modulatory role in ARDS-like conditions (Huang et al., 2025).

## The clinical impact of oxidative stress on ARDS

The pathophysiological effects of oxidative stress translate into significant clinical consequences for ARDS patients.

Impaired oxygenation and hypoxemia: In cases of ARDS, the activation of neutrophils and elevated levels of inhaled oxygen lead to a rise in ROS, resulting in oxidative stress (Pacht et al., 1991). The critically ill are particularly susceptible to redox imbalance, which can exacerbate the pathophysiological processes, leading to ARDS (Gutteridge and Mitchell, 1999). In the context of resuscitation, exposure to 100% oxygen has been shown to cause intestinal glutathione oxidation and reoxygenation injury, highlighting the detrimental effects of oxidative stress on tissue function (Haase et al., 2004). An elevation in ROS contributes to increased cellular damage and impedes proper gas exchange in the lungs, which ultimately lowers the PaO_2_/FiO_2_ ratio (Gu et al., 2022). Alda-1 treatment attenuates hyperoxia-induced ALI by enhancing antioxidant defenses (Sidramagowda Patil et al., 2020). For patients with ARDS, oxygen saturation levels are critical indicators for gauging the seriousness of the illness, with oxidative stress being a major contributing factor in this regard.

Multiple studies have demonstrated a strong correlation between oxidative stress markers and the severity of ARDS. These markers are increasingly recognized for their potential as prognostic indicators (Klouda and Stone, 2020). In patients with ARDS, reduced total thiol levels, along with increased ferritin and LDH levels, reflect the significant role played by oxidative stress and inflammation. These biomarkers offer a relatively fast and cost-effective method for assessing ARDS progression. Their prognostic value is further supported by high area under the curve (AUC) values reported in related studies (Martinez Mesa et al., 2021). Increased levels of oxidative stress markers, such as MDA and total oxidant status (TOS), together with inflammatory markers like IL-6 and CRP, have been associated with poor outcomes in severe COVID-19 cases (Coronel et al., 2023). Moreover, studies have shown elevated hypoxanthine in non-survivors with ARDS. This increase is associated with greater oxidative stress and higher mortality risk (Yu et al., 2020).

### Reduced antioxidant levels

Activated leukocytes in the pulmonary microvasculature release ROS, which not only contribute to tissue injury but also lead to the exhaustion of antioxidants (Ronchi et al., 2012). This oxidative damage creates a vicious cycle where increased ROS levels further deplete antioxidant reserves, exacerbating lung injury and inflammation. A clinical cohort study demonstrated that individuals with ARDS have significantly lower levels of non-enzymatic antioxidants, such as vitamin C and ubiquinol-10. This reduction is consistent with increased oxidative stress in these patients (Ciapetti et al., 2018). Likewise, other clinical studies have shown that patients with ARDS present lower plasma concentrations of key antioxidants. These include α-tocopherol, β-carotene, and selenium. Such findings suggest a weakened ability to counteract oxidative stress in ARDS (Nelson et al., 2003; Metnitz et al., 1999). SOCS-1 gene transfer enhances antioxidant defenses, protecting against hyperoxic ALI (Galam et al., 2015).

### Protein and lipid oxidation

In ARDS, the inactivation of lung surfactant is heavily influenced by the oxidation of surfactant protein B (SP-B) (Sarker et al., 2011). When tryptophan in SP-B undergoes oxidation, it partially disrupts its α-helical form and changes how it interacts with lipids, a key factor for sustaining surfactant activity. Surfactant protein A (SP-A) is also subject to oxidative alterations, particularly through the nitration and chlorination of its tyrosine residues (Davis et al., 2002). This alteration prevents SP-A from clustering lipids and attaching to mannose, both of which are crucial for its role in defending the host. The disruption of surfactant function in ARDS is also connected to lipid oxidation, as the presence of oxidized lipids can change the surface tension dynamics of the surfactant, ultimately affecting lung functionality (Chen et al., 2013). ACO1 overexpression exacerbates oxidative damage in lung vasculatures (Fukumoto et al., 2022).

## Oxidative stress biomarkers

Based on insights gathered from numerous studies, oxidative stress markers can largely be divided into the following categories. The principal biomarkers of oxidative stress in ARDS are illustrated and compared in Table 1.

**TABLE 1:** Comparison of oxidative stress biomarkers in ARDS.

Category	Biomarker	Source & Generation	Biological Role & Mechanism	Clinical/Prognostic Significance	Representative References (PMID)
Markers of Oxidative Damage	MDA	End-product of peroxidation, primarily ROS-mediated attack on PUFAs in cell and mitochondrial membranes.	Classic indicator of lipid peroxidation, reflecting oxidative damage to membranes. Elevated levels linked to OS and inflammation.	Significantly elevated in ALI models (e.g., LPS, sepsis). Reduction used to assess antioxidant therapy efficacy (e.g., quercetin).	36982166, 32161620
	8-isoprostane	Generated via non-enzymatic peroxidation of arachidonic acid by ROS.	Gold standard for in vivo OS, an early marker of lipid peroxidation.	Early biomarker in chlorine-induced lung injury; elevated in ARDS serum/BALF, correlates with severity.	9872202, 21824465, 29017959
	AOPPs	Cross-linked polymers of plasma proteins (mainly albumin) formed under OS.	Reflects protein oxidative damage; activates neutrophils/monocytes, promoting cytokine release.	Elevated early in post-cardiac surgery ALI, an independent predictor of ALI.	27026925
	8-OHdG	Major product of oxidative damage to nuclear and mitochondrial DNA by hydroxyl radicals and ROS.	Marker of DNA oxidative damage; accumulation leads to mutations and apoptosis.	Elevated in ALI/ARDS, correlates with severity and ventilation dependency.	19506468, 36437551
Antioxidant Defense System	SOD	Endogenous enzyme clearing superoxide anions, located in mitochondria and cytoplasm.	First line of antioxidant defense; decreased activity indicates impaired ROS clearance.	Decreased in ARDS/ALI; therapies (e.g., genipin) increase activity to protect lungs.	35305560, 39629132, 26544923
	CAT	Endogenous enzyme clearing hydrogen peroxide, primarily in mitochondria and cytoplasm.	Part of antioxidant defense; reduced activity exacerbates OS.	Decreased in ARDS; enhancing activity reduces lung injury.	35764933, 24486341
	GSH	Non-enzymatic antioxidant, scavenges ROS via GPx, oxidized to GSSG.	Key intracellular antioxidant; GSH/GSSG ratio reflects redox state.	Depleted in ARDS alveolar fluid; supplementation (e.g., uridine) mitigates mitochondrial damage.	35305560, 37301835, 37121999
	HO-1	Stress-inducible enzyme activated via Nrf2 pathway.	Degrades heme into antioxidant/anti-inflammatory products (CO, bilirubin).	Lower serum levels linked to better ARDS survival; upregulated by therapies (e.g., quercetin).	33653364, 33774474, 34049949
	Trx	Ubiquitous redox-regulating protein.	Regulates protein redox state, inhibits OS and apoptosis.	Serum levels correlate with ALI severity post-TBI; involved in protective mechanisms.	28347674, 24295151
	GPX4	Selenoprotein reducing lipid hydroperoxides, located in mitochondria and cytoplasm.	Prevents lipid peroxidation and ferroptosis; key antioxidant in ALI.	Downregulated in ferroptosis-driven ALI; restored by therapies (e.g., quercetin).	32161620, 35305560, 36913799
Markers of Inflammation, Cell Injury & Specificity	MPO	Stored in neutrophil azurophilic granules, released upon activation.	Marker of neutrophil activation; produces oxidants (e.g., HOCl), worsening tissue damage.	Elevated in ALI/ARDS BALF/lung tissue, indicates neutrophilic inflammation.	33751359, 34635643, 37841910
	Ferritin	Intracellular iron-storage protein, released as an acute-phase reactant.	Regulates iron metabolism; excess iron catalyzes ROS via Fenton reaction, linked to ferroptosis.	Elevated in ARDS (e.g., COVID-19), predicts severity; Fth1hi neutrophils aggravate ALI.	35525119, 37880599, 38148147
	mtDNA	Released into cytoplasm/circulation after ROS-induced mitochondrial damage.	DAMP activating STING-NLRP3 inflammasome, triggers pyroptosis/inflammation.	Higher in ARDS vs. controls, correlates with mortality and mitochondrial dysfunction.	35401830, 34218252, 33252860
	HMGB1	Nuclear protein released during cell death/stress, acts as a DAMP.	Binds TLR4/RAGE, promotes inflammation.	Elevated in ARDS, linked to severity; inhibited by therapies (e.g., danlou tablet).	19506468, 32432771, 34703269
	SLC7A11	Component of system xc- cystine/glutamate antiporter, on cell membranes.	Imports cystine for GSH synthesis, protects against OS; downregulation triggers ferroptosis.	Decreased in ALI ferroptosis models; targeted by therapies (e.g., menaquinone-4).	*35305560*, *37880599*, 32161620
	CC16	Secreted by club cells in the lung.	Protects lung epithelium; reflects epithelial injury.	Decreased in polytrauma ALI BALF/plasma, potential biomarker for lung damage.	35596754, 28548310

Abbreviations: ALI: Acute Lung Injury; AOPPs: Advanced Oxidation Protein Products; ARDS: Acute Respiratory Distress Syndrome; BALF: Bronchoalveolar Lavage Fluid; CAT: Catalase; CC16: Club Cell Protein 16; DAMP: Damage-Associated Molecular Pattern; Fth1: Ferritin Heavy Chain 1; GPX4: Glutathione Peroxidase 4; GSH: Glutathione (Reduced); GSSG: Glutathione Disulfide (Oxidized); HMGB1: High Mobility Group Box 1; HO-1: Heme Oxygenase-1; HOCl: Hypochlorous Acid; LPS: Lipopolysaccharide; MDA: Malondialdehyde; MPO: Myeloperoxidase; mtDNA: Mitochondrial DNA; NLRP3: NOD-like Receptor Protein 3; Nrf2: Nuclear Factor Erythroid 2-Related Factor 2; 8-OHdG: 8-Hydroxy-2'-Deoxyguanosine; OS: Oxidative Stress; PUFAs: Polyunsaturated Fatty Acids; RAGE: Receptor for Advanced Glycation Endproducts; ROS: Reactive Oxygen Species; SLC7A11: Solute Carrier Family 7 Member 11; SOD: Superoxide Dismutase; STING: Stimulator of Interferon Genes; TBI: Traumatic Brain Injury; TLR4: Toll-Like Receptor 4; Trx: Thioredoxin.

### DNA damage biomarkers

8-hydroxy-2-deoxyguanosine (8-OHdG) is an established marker for oxidative damage to DNA, and it plays a role in lung-related diseases (Graille et al., 2020a). Elevated levels of circulating mitochondrial DNA (mtDNA), a commonly assessed indicator of mitochondrial dysfunction, are notably higher in patients with ARDS than in those without the condition (Mcclintock et al., 2022).

### Lipid peroxidation biomarkers

F2-isoprostanes, malondialdehyde (MDA), and 8-isoprostane are extensively studied as biomarkers for lipid oxidation (Graille et al., 2020b). Utilizing gas chromatography and mass spectrometry (GC-MS) to analyze exhaled air has enabled the identification of potential biomarker signatures of ARDS, such as succinic acid, where most of the identified indicators are related to lipid peroxidation processes (Horvath et al., 2017).

### Protein oxidation biomarkers

The detection of carbonyl groups, protein semialdehyde, and methionine sulfoxide in proteins reflects oxidative modifications and is related to a range of diseases, including those related to the lung (Dalle-Donne et al., 2003). Advanced oxidation protein products (AOPPs) also serve as markers (Elkabany et al., 2020).

### Antioxidant enzyme levels

Beyond that, research efforts have explored the possibility of utilizing antioxidant enzymes, especially superoxide dismutase (SOD) and catalase, as potential biological indicators, as both have shown associations with the prognosis of ARDS (Frijhoff et al., 2015).

Levels of AOPPs and 8-OHdG levels correlate with ARDS severity and mechanical ventilation dependence and can serve as early predictors for evaluating treatment outcomes (Elkabany et al., 2020). Meanwhile, research findings suggested that single nucleotide polymorphisms (SNPs) in antioxidant enzymes, such as GSTP1, MnSOD, and eNOS, significantly influence oxidative stress biomarker concentrations in patients with ARDS (Elkabany et al., 2020). With the rise of precision medicine, personalized approaches based on oxidative stress biomarkers are expected to emerge as a novel strategy for managing ARDS (Spadaro et al., 2019). This method aims to address ARDS heterogeneity by identifying specific patient sub-phenotypes that may respond differently to various treatments. Modifying antioxidant therapy based on biomarker levels could reduce unnecessary treatments and side effects, thereby enhancing therapeutic outcomes (Dragoi et al., 2024; Reilly et al., 2018). However, the lack of standardized protocols and validation for oxidative stress biomarkers presents a challenge for their clinical implementation (Marrocco et al., 2017).

## Oxidative stress and prognosis of ARDS

The relationship between oxidative stress biomarkers and patient outcomes provides valuable prognostic information that can guide clinical decision-making. The elevation of oxidative stress levels is typically associated with the disease course and prognosis of ARDS. Studies have shown a significant correlation between the concentration of oxidative stress biomarkers and the prognosis of ARDS. High levels of ROS and oxidative stress markers are closely associated with prolonged hospital stays, increased dependence on mechanical ventilation, higher ICU admission rates, and elevated mortality (Quinlan et al., 1997). A meta-analysis indicates antioxidants may reduce ICU stay but not mortality, highlighting inconsistent efficacy (Bo et al., 2021).

Circulating mtDNA has emerged as a key oxidative stress biomarker in ARDS. Higher blood levels of mtDNA are strongly correlated with ARDS when compared to non-ARDS controls, suggesting its potential involvement in the disease’s pathophysiological processes (Mcclintock et al., 2022). ARDS patients with lower baseline levels of heme oxygenase-1 (HO-1) tend to have better survival rates. Moreover, serum HO-1 levels are associated with clinical parameters such as serum bilirubin, LDH, and surfactant protein-D, all of which are indicators of disease severity and oxidative stress (Nagasawa et al., 2020). Biomarkers such as total thiol, ferritin, and LDH have been examined for their potential to predict the severity of COVID-19 and aid in clinical decision-making (Martinez Mesa et al., 2021). The detection of oxidative markers like chlorotyrosine and nitrotyrosine in bronchoalveolar lavage fluid (BALF) may reflect oxidative stress levels and could be useful in predicting both the onset and progression of ARDS (Lenz et al., 1999; Sittipunt et al., 2001).

Changes in serum free fatty acid ratios, particularly increases in unsaturated fatty acids like oleate and linoleate, can predict the development of ARDS in at-risk patients (Quinlan et al., 1996). In those with ARDS, isoprostanes—markers of lipid peroxidation—are considerably elevated in exhaled breath condensate (Carpenter et al., 1998). This suggests a rise in oxidative stress and lipid peroxidation, enabling a non-invasive method to assess oxidant stress in ARDS. Monitoring oxidative stress in patients with ARDS through the examination of exhaled gases, such as NO and H_2_O_2_, provides an effective means of real-time evaluation. This approach exhibits a significant correlation with patients’ clinical manifestations and the trajectory of disease progression (Liu et al., 2015; De Broucker et al., 2012).

Although investigations into the roles of MDA, SOD, and glutathione (GSH) specifically within ARDS remain sparse, their established utility in other pathological contexts suggests they hold promise for assessing disease development and informing tailored therapeutic strategies. Furthermore, diminished serum concentrations of antioxidant enzymes, including SOD and catalase, have been implicated in the worsening of various diseases, while heightened levels of ROS by-products, such as MDA and 4-hydroxy-2-nonenal (4-HNE), demonstrate a robust association with elevated mortality rates (Metnitz et al., 1999; Leff et al., 1993).

## Oxidative stress and multi-organ dysfunction

The systemic nature of oxidative stress in ARDS often leads to multi-organ dysfunction, complicating patient management and outcomes. Patients diagnosed with ARDS commonly face dysfunction in other vital organs like the kidneys, heart, and liver. The significance of oxidative stress in these organ malfunctions has become a topic of increasing interest.

### Renal dysfunction

Oxidative stress plays a critical role in mediating acute kidney injury (AKI) in patients with ARDS through several interconnected mechanisms: endothelial dysfunction, mitochondrial damage, immune activation, and cellular injury (Singh et al., 2024). Singh et al. demonstrated a correlation between the incidence of AKI in ARDS and elevated levels of oxidative stress markers (Singh et al., 2024). The kidney is particularly sensitive to oxidative stress due to the generation of ROS and reactive nitrogen species (RNS) from sources like mitochondria and NOX (Tomsa et al., 2019). Additionally, ARDS-related treatments may adversely impact kidney function. Ruan et al. noted that while inhaled nitric oxide can improve oxygenation in ARDS patients, it is also associated with an increased risk of kidney dysfunction, particularly at higher dosages and among the elderly (Ruan et al., 2016).

### Cardiac dysfunction

Cardiac dysfunction is often seen as a complication during ARDS, where oxidative stress causes an overload of intracellular calcium, which seems to be a key factor in the impairment of myocytes (Dhalla et al., 2022). Studies have also linked impaired cardiac function indicators in ARDS patients with increased ROS levels (Dhalla et al., 2022; Senoner and Dichtl, 2019). Oxidative stress plays a role in the progression of arrhythmias and heart failure, impacting the heart’s electrical properties by modifying ion channels and signaling pathways, which can result in arrhythmogenic conditions (Senoner and Dichtl, 2019). Research has shown that targeting the TLR3/4 and NLRP3/NF-κB pathways can lead to a decrease in oxidative stress and provide anti-inflammatory benefits in a mouse model of ARDS induced by LPS/POLY I:C, ultimately enhancing the functionality of both the lungs and heart (Jain et al., 2023).

### Hepatic injury

Oxidative stress also affects liver function in ARDS patients. Insufficient GSH and its elevated usage in individuals with liver impairment may be factors in the adverse results associated with ARDS, given that the lungs rely on the liver for GSH supply (Foreman et al., 2002). Liu et al. revealed that in patients who developed ARDS after orthotopic liver transplantation, levels of inflammatory mediators such as TNF-α and IL-8, as well as oxidative stress markers like MDA, NO, H_2_O_2_, and 8-iso-prostaglandin F2α, were significantly higher compared to those who did not develop ARDS (Liu et al., 2015).

## Therapeutic targeting of oxidative stress in ARDS

Given the central role of oxidative stress in the pathogenesis of ARDS, therapeutic strategies aimed at inhibiting ROS production or enhancing antioxidant defenses are being actively explored. Therapeutic strategies for inhibiting oxidative stress involve the use of antioxidants, modulation of intracellular antioxidant enzyme activity, and reduction of ROS production, all aimed at mitigating lung damage. The application and comparison of common antioxidants in ARDS are presented in Table 2.

**TABLE 2:** Comparison of antioxidant therapies in ARDS: mechanisms, dosage, and clinical applications.

Antioxidant	Clinical Trial Stage	Mechanism of Action	Dosage & Administration (Examples)	Clinical/Preclinical Efficacy & Limitations	References (PMID)
Vitamin C (Ascorbic Acid)	Phase II/III, Retrospective, Observational	Direct ROS scavenger; Recycles Vitamin E; Enhances endothelial barrier function and immune cell activity; Reduces inflammation via NF-κB inhibition.	High-dose IV (e.g., 50 mg/kg q6h or 6 g/day).	Conflicting clinical evidence. The CITRIS-ALI trial showed no improvement in primary outcomes but suggested mortality benefit in secondary analysis. Reduces MDA, and IL-8 in TRALI trial; improves oxygenation and reduces mortality in some COVID-19 studies. Limited by potential oxalate nephropathy risk at high doses.	31573637, 34866234, 36144269
Vitamin E (α-Tocopherol)	Phase II, Preclinical	Lipophilic antioxidant; Protects cell membranes from lipid peroxidation; Reduces NF-κB activation.	High enteral/oral dosages (e.g., 300-800 IU/day); IV in some preclinical models.	Moderate preclinical evidence; improves lung function and reduces OS. Limited clinical data; no strong mortality benefit in ARDS. Effective in combination with Vitamin C or Se.	6756260, 12665164, 33432848
N-acetylcysteine (NAC)	Phase III, Preclinical	GSH precursor; Direct ROS scavenger; Modulates Nrf2 pathway; Reduces inflammation via NF-κB inhibition.	IV (e.g., 70 mg/kg q8h), inhaled, or oral administration.	Replenishes GSH effectively; RCTs show reduced ALI duration and improved cardiac index, but inconsistent mortality benefits. Limited efficacy for phosgene-induced ALI when inhaled.	9228372, 20384467, 34114174, 1617983
Selenium (Se)	Phase III, Preclinical	Cofactor for GPx, neutralizing H₂O₂ and lipid hydroperoxides; Enhances antioxidant enzyme activity.	IV as sodium selenite (e.g., 1 mg/kg in preclinical models).	Improves plasma Se and GPx activity; may enhance lung function but lacks consistent survival benefits in clinical trials. Porous Se@SiO2 nanospheres show promise in PQ-induced ALI.	30001171, 34487287, 40114196
Coenzyme Q10 (CoQ10)	Preclinical	Mitochondrial-targeted antioxidant; Scavenges lipid peroxyl radicals; Modulates NF-κB and MAPK signaling; Reduces oxidative/nitrative stress.	Preclinical oral/IP doses (e.g., 5-100 mg/kg).	Strong preclinical evidence; protects against sepsis/LPS-induced ALI by preserving mitochondrial function and reducing inflammation. Limited human data; poor bioavailability a challenge, mitigated by targeted forms like MitoQ.	39191119, 34280918
Resveratrol	Preclinical	Inhibits NF-κB, MAPK, and TXNIP/NLRP3 inflammasome; Activates SIRT1 and Nrf2 pathways.	Preclinical (oral, IP, intranasal; e.g., 10-50 mg/kg).	Reduces lung edema, inflammation, and OS in ALI models (LPS, I/R, endotoxemia). Limited by low bioavailability; clinical translation pending.	36091774, 33722710
Curcumin	Preclinical	Inhibits NF-κB, MAPK, and NLRP3 inflammasome; Activates Nrf2 pathway; Reduces oxidized proteins; Inhibits ASK1.	Preclinical (oral, aerosolized; e.g., 50-200 mg/kg).	Reduces inflammation, OS, and lung injury in ALI models (LPS, sepsis). Limited clinical data; poor solubility/bioavailability improved by aerosolized forms.	37700323, 35106369, 39342264

Abbreviations: ALI: Acute Lung Injury; ARDS: Acute Respiratory Distress Syndrome; ASK1: Apoptosis Signal-regulating Kinase 1; COVID-19: Coronavirus Disease 2019; GPx: Glutathione Peroxidase; GSH: Glutathione (Reduced); IL-8: Interleukin-8; IP: Intraperitoneal; IV: Intravenous; LPS: Lipopolysaccharide; MAPK: Mitogen-Activated Protein Kinase; MDA: Malondialdehyde; NAC: N-acetylcysteine; NF-κB: Nuclear Factor kappa-light-chain-enhancer of activated B cells; NLRP3: NOD-like receptor protein 3; Nrf2: Nuclear factor erythroid 2-related factor 2; OS: Oxidative Stress; RCT: Randomized Controlled Trial; ROS: Reactive Oxygen Species; SIRT1: Sirtuin 1; TRALI: Transfusion-Related Acute Lung Injury; TXNIP: Thioredoxin-Interacting Protein.

### Application of antioxidants

Antioxidants alleviate oxidative damage by scavenging excess ROS, thereby improving lung function. Commonly studied antioxidants include N-acetylcysteine (NAC), vitamin C, vitamin E, Prelox, etc. by eliminating surplus ROS, antioxidants can abate oxidative stress, a critical factor in the emergence of acute lung injury (ALI). This reduction in ROS can subsequently help lessen inflammation and protect lung tissue from injury (Liu et al., 2021; Cen et al., 2021). The administration of Vitamin C, E, and α-Lipoic Acid in a rat model of ARDS caused by oleic acid has been shown to diminish cytokine levels and increase the activity of antioxidant enzymes. Additionally, these compounds alleviated lung tissue injury (Erol et al., 2019). Vitamin C and sulforaphane, both recognized for their antioxidant properties, have been identified as enhancers of macrophage function by diminishing the concentration of harmful proteins, including HMGB1, which contributes to reducing lung damage from high oxygen concentrations (Patel et al., 2020). By activating the Nrf2 pathway, 4-octyl itaconate and mitoQ enhance the expression of protective genes that combat oxidative damage and inflammation, emphasizing their promising role in the therapy of ALI (Cen et al., 2021; Zheng et al., 2019). Similarly, SS31, a mitochondria-targeted antioxidant peptide, has been shown to inhibit NLRP3 inflammasome activation and pyroptosis by regulating S100A8, offering a novel therapeutic approach to mitigate LPS-induced ALI (Luo et al., 2024). Fe-curcumin nanoparticles leverage nanozyme activity to scavenge ROS and suppress NLRP3 inflammasomes, demonstrating efficacy in ALI treatment through dual anti-inflammatory and antioxidant effects (Yuan et al., 2022). Moreover, syringin has been found to alleviate ROS-induced ALI in A549 lung epithelial cells by activating the SIRT1/STAT6 pathway to inhibit ferroptosis, presenting a new antioxidant strategy targeting programmed cell death (Cai et al., 2025). Lai et al. demonstrated that uridine supplementation reduces sepsis-induced ALI by inhibiting macrophage ferroptosis through Nrf2 activation, offering a novel antioxidant approach (Lai et al., 2023). Additionally, Qin et al. developed a Keap1-Nrf2 inhibitor (compound 1c) that significantly reduces oxidative stress and inflammation in LPS-induced ALI, highlighting a promising targeted therapy (Qin et al., 2025). Aspirin-triggered resolvin D1 enhances resolution of hyperoxic ALI, reducing ROS-driven inflammation (Cox et al., 2015).

However, clinical studies have shown that antioxidants yield inconsistent results. Parenteral NAC treatment started within 8 h of diagnosis increases the intracellular GSH in the granulocytes of ARDS patients without decreasing spontaneous oxidant production by these cells (Laurent et al., 1996). In an RCT, among critically ill individuals with transfusion-related acute lung injury (TRALI), the use of high-dose vitamin C was linked to a significant reduction in oxidative stress (MDA), decreased pro-inflammatory IL-8 and CRP levels, and an increase in the anti-inflammatory marker IL-10 (Kassem et al., 2022). In another RCT, while selenium did not significantly influence the survival rates, length of mechanical ventilation, or duration of ICU stay for patients with ARDS, it successfully elevated lung antioxidant levels, reduced inflammation, and enhanced lung function (Mahmoodpoor et al., 2019). In particular groups of septic patients, especially those with ARDS indicated by a low Carrico index, selenium might be beneficial (Kocan et al., 2014). Still, the definitive effect of this element on mortality and other health outcomes in cases of sepsis has not been firmly determined. The reasons for failure in some trials are multifaceted, often involving inadequate dosing, improper timing of administration relative to disease stage, or routes of administration that do not achieve optimal bioavailability at the target tissue. Furthermore, dose-dependent effects are critical, as too low a dose may be ineffective, while excessively high doses could potentially lead to adverse effects or even pro-oxidant activities.

### Potential of other oxidative stress inhibitors

Other agents that effectively reduce oxidative stress are also under investigation, including novel small-molecule antioxidants and natural plant extracts. In an *in-vitro* experiment, investigators successfully isolated two new and seven known compounds from the methanol extract of Dendrobium virgineum. Among these, 2-methoxy-9,10-dihydrophenanthrene-4,5-diol (referred to as compound 3) exhibited the most significant protective effects against oxidative stress induced by H_2_O_2_ in ARPE-19 cells (Panuthai et al., 2023). Antia, an antioxidant obtained from natural sources like the yamabushitake mushroom, improves antioxidant protection by increasing GSH levels and diminishing MDA (El Sayed and Ghoneum, 2020). Uridine supplementation reduces lung injury, inflammation, tissue iron levels, and lipid peroxidation by modulating the expression of ferroptosis biomarkers such as SLC7A11, GPX4, and HO-1 while restricting lipid synthesis gene ACSL4 (Lai et al., 2023). Histone deacetylase 3 (HDAC3) deficiency offers a protective effect against ALI by enhancing mitochondrial quality control and reducing inflammation, thereby maintaining epithelial barrier integrity (Li et al., 2023). Molybdenum nanodots (MNDs) have emerged as a promising nanomaterial, exhibiting strong ROS scavenging capabilities and inhibiting NLRP3-dependent pyroptosis, significantly ameliorating ALI in mice (Yan et al., 2023). BMI1 silencing exacerbates mitochondrial dysfunction in hyperoxic ALI, suggesting targeted mitochondrial therapies (Hernandez-Cuervo et al., 2022).

Some antioxidant regimens have demonstrated promise in the clinical treatment of ARDS. In an RCT, the oral adjunct therapy Beta-hydroxybutyrate has exhibited favorable effects in improving the outcomes of COVID-19-related ARDS, notably by decreasing inflammation, oxidative stress, and levels of fatigue among patients (Shahtaghi et al., 2024). While the enteral-feeding diet with eicosapentaenoic acid, gamma-linolenic acid, and antioxidants did not lower oxidative stress levels over a period of at least 4 days, it successfully normalized plasma concentrations of beta-carotene and alpha-tocopherol, seemingly offering protection against additional lipid peroxidation in ARDS patients (Nelson et al., 2003). Early administration with antioxidant supplements, including alpha-tocopherol and vitamin C, was reported to help decrease the likelihood of developing ARDS and hospital-acquired pneumonia, and it also shortened the length of ICU stay for critically ill surgical patients (Nathens et al., 2002). Likewise, research outcomes, both experimental and clinical, revealed that administering vitamin E, especially in high doses, might confer protective effects in ARDS treatment by alleviating oxidative stress and improving lung function (Wolf and Seeger, 1982).

### Challenges and limitations of antioxidant therapy

While there is evidence that antioxidant therapy can be effective in animal models of ARDS, the clinical results have been inconsistent, and numerous challenges remain.

#### Limitations of clinical research

Despite the theoretical benefits of antioxidants in diminishing oxidative stress, the results from clinical trials have often fallen short of what was anticipated. This inconsistency may stem from factors including patient heterogeneity (e.g., genetic polymorphisms like GSTP1 influencing N-acetylcysteine (NAC) efficacy (Wang X. et al., 2022; Moradi et al., 2009), timing of intervention, dosage, and route of administration. A study demonstrated that both NAC and procysteine effectively increased red blood cell glutathione levels over a 10-day treatment period, suggesting a potential therapeutic role in managing oxidative stress in ARDS patients (Bernard et al., 1997). Though the duration of ALI was reduced, and there was a notable increase in cardiac index in patients treated with NAC or procysteine, there was no significant difference in mortality rates among the treatment and placebo groups (Bernard et al., 1997). In a pilot study, the application of NAC in individuals with ARDS due to COVID-19 failed to decrease death rates, the length of time on mechanical ventilation, or the duration of hospital stays (Taher et al., 2021). Factors contributing to these discrepancies may include patient heterogeneity, timing of intervention, dosage, and route of administration.

#### Dual role of oxidative stress

In ARDS, oxidative stress is not solely harmful; it also plays an essential role in immune responses and cellular repair processes. ROS and RNS are vital components in the regulation of the immune system, affecting how immune responses are initiated and resolved, including pathogen clearance. Over-suppression of oxidative stress may interfere with the body’s normal immune defense, potentially increasing the risk of infection. Therefore, precisely modulating oxidative stress without completely inhibiting it remains a critical issue in clinical treatment (Mullen et al., 2020).

#### Development of novel antioxidants

Many traditional antioxidants have limited efficacy in clinical treatment, potentially due to poor bioavailability, instability, or inadequate targeting. Therefore, there is a need to develop more effective novel antioxidants. Emerging therapies, such as targeted antioxidant enzymes and nanotechnology-based drug delivery systems, are becoming research hotspots. For example, PECAM-1 targeted delivery of SOD leverages nanotechnology to enhance drug delivery to endothelial cells, potentially reducing side effects and improving therapeutic outcomes (Hood et al., 2012). Biopolymer nano-based drug delivery systems with antioxidative properties represent a rapidly evolving field with promising applications. These systems offer enhanced drug delivery capabilities, reduced side effects, and antioxidative benefits, making them a valuable tool in treating oxidative stress-related diseases (Stevanovic and Filipovic, 2024).

#### Ethical considerations in antioxidant trials

Ethical concerns, such as high-dose antioxidant safety and racial differences in oxidative stress responses, further complicate trial design (Mullen et al., 2020). Clinical trials involving antioxidants must navigate several ethical considerations. The safety of high-dose antioxidant administration needs careful evaluation, as supra-physiological doses can sometimes exert pro-oxidant effects or interfere with essential physiological ROS signaling. Ensuring population diversity in trials is crucial, as responses to oxidative stress and antioxidants can vary across different racial and ethnic groups. More research is needed to understand how these differences might affect therapeutic outcomes and to ensure equitable benefit from antioxidant strategies.

#### Targeted management of oxidative stress and immune responses

Given the complex interaction between oxidative stress and immune responses, future therapeutic approaches may need to involve not just antioxidant therapies but also a more holistic strategy to modulate the delicate balance between these two processes. For instance, the combination of N-acetyl lysyl-tyrosyl-cysteine amide (KYC) and 4-aminobenzoic acid hydrazide (ABAH) has proven effective in reducing oxidative stress by targeting MPO activity in related diseases (Chen et al., 2020). Furthermore, PECAM-targeted delivery of SOD has been shown to significantly reduce the expression of inflammatory markers, such as vascular cell adhesion molecule-1 (VCAM-1), in response to cytokines like TNF and IL-1β, suggesting a marked attenuation of endothelial pro-inflammatory activation (Howard et al., 2014). The use of immuno-liposomes encapsulating NOX inhibitors, such as MJ33, targeted to platelet endothelial cell adhesion molecule (PECAM-1), has shown effectiveness in specifically binding to endothelial cells. This combined approach significantly reduces angiotensin-induced ROS production both *in vitro* and *in vivo*, highlighting its potential in managing oxidative stress (Hood et al., 2012).

## Future directions and challenges

To advance our understanding and treatment of ARDS, future research must address both the mechanistic complexities of oxidative stress and the practical challenges of clinical translation.

### Mechanistic elucidation

Oxidative stress is a central player in the onset and progression of ARDS, yet its specific mechanisms and interactions with other pathways are not fully elucidated. Future studies should focus on delineating the precise molecular pathways linking oxidative stress to ARDS pathogenesis. Such investigations could uncover novel therapeutic targets and provide a more comprehensive understanding of the disease’s underlying pathophysiology.

### Improving therapeutic efficacy and targeting

Antioxidant therapies often fail clinically due to non-specific ROS suppression, which disrupts immune defenses, and poor delivery to target tissues (Garcia-Sanchez et al., 2020; Forman and Zhang, 2021). Novel approaches, such as endothelial-targeted nanoparticles (e.g., PECAM-1 delivery (Hood et al., 2012)), mitochondrial-specific antioxidants (e.g., mitoQ (Cen et al., 2021)), or ferroptosis inhibitors (e.g., uridine (Lai et al., 2023)), could improve efficacy. Key questions include whether targeting ferroptosis is superior to broad antioxidants and how to balance ROS modulation with immune function.

### Clinical validation and collaboration

Multicenter clinical trials are essential to validate the efficacy and safety of antioxidant therapies across diverse populations. A meta-analysis of treatments like N-acetylcysteine (NAC), selenium, and vitamin C suggests potential benefits in improving clinical outcomes, yet these therapies have not consistently reduced all-cause mortality and may pose risks in low-risk patients (Bo et al., 2021). Larger, more diverse trials, potentially incorporating biomarker or genetic stratification (e.g., considering GSTP1 polymorphisms for NAC) (Moradi et al., 2009) are needed to assess their universal applicability and variability in patient responses. Strengthened international collaboration and data sharing are crucial for accelerating progress.

### Biomarker development and monitoring

A key challenge lies in effectively monitoring oxidative stress levels to inform treatment decisions. While oxidative stress biomarkers hold potential for diagnosing ARDS and assessing its prognosis, their clinical utility is hampered by several limitations. Current biomarkers often lack sufficient specificity and sensitivity for ARDS, as many markers are altered in various critical illnesses (Lopez et al., 2013). Enhancing their specificity and validating their utility for guiding therapy remains a critical area for future research (Quinlan et al., 1997). Given the rapid progression of ARDS, real-time or frequent tracking of oxidative stress biomarkers is desirable for adjusting treatment strategies promptly. However, achieving this capability and integrating biomarker data with clinical signs, imaging, and other assessments into a multidimensional predictive framework remains a significant unresolved challenge (Frijhoff et al., 2015).

## Conclusion

Oxidative stress plays a key role in the pathogenesis of ARDS, causing cellular damage, inflammation, and organ dysfunction. Excess ROS damage cell membranes, activate inflammatory pathways, and disrupt the alveolar-capillary barrier, leading to pulmonary edema and respiratory failure. ROS also induce PCD like apoptosis, pyroptosis, or ferroptosis, and mitochondrial dysfunction, worsening tissue injury. Although the detrimental role of oxidative stress in ARDS is well recognized, inconsistent findings from clinical studies pose challenges for treatments aimed at inhibiting ROS. Future research should identify novel therapeutic targets by further exploring their molecular mechanisms and interactions. Developing targeted delivery systems and strategies for precisely modulating oxidative stress could enhance clinical effectiveness, and the application of customized or combined therapies might advance the management of ARDS. Ultimately, targeting oxidative stress offers promise for treating ARDS and other acute lung injuries, but significant hurdles in clinical translation must be overcome.
